# Environment impact and probabilistic health risks of PAHs in dusts surrounding an iron and steel enterprise

**DOI:** 10.1038/s41598-021-85053-4

**Published:** 2021-03-24

**Authors:** Xiaofeng Wei, Chun Ding, Chunzhu Chen, Li Zhu, Guiqin Zhang, Youmin Sun

**Affiliations:** 1 School of Municipal and Environmental Engineering, Shandong Jianzhu University, Jinan, 250101 Shandong China; 2 Clear Science and Technology Company Limited, Jinan, 100029 Beijing China

**Keywords:** Environmental sciences, Environmental social sciences

## Abstract

Dust can be regarded as environmental medium that indicates the level and spatial distribution of polycyclic aromatic hydrocarbons (PAHs) coming from different pollution sources. In this study, samples including road dust, roof dust, and bare soil near an iron and steel enterprise (ISE) in Laiwu city of North China were collected. To assess the environment impact, atmosphere particulates and one flue dust from a coking plant were simultaneously sampled. Sixteen USEPA PAHs were detected quantitatively by Gas Chromatography Mass Spectrometry (GC–MS). A laser particle size analyzer was used to obtain the grain size of the dust particle samples. The results showed that PAH concentrations displayed great variability in the dust samples. The ∑_16_PAHs concentration was found to be between 0.460 and 46.970 μg/g (avg ± sd 10.892 ± 1.185 μg/g) in road dust, between 0.670 and 17.140 μg/g (avg ± sd 6.751 ± 0.692 μg/g) in roof dust, and 13.990 ± 1.203 μg/g in bare soil. In the environment atmosphere sites, the ∑_16_ PAHs value in PM_2.5_ constituted a very large proportion of PM_10_, indicating that PAHs in finer particle sizes should be given greater emphasis. The ∑_16_PAHs concentration was relatively high in the area close to the ISE because of the great impact of the ISE industrial activities. PAH concentration curves were similar, and the most abundant individual PAHs in the atmosphere sites were BbF, BkF, and Flu, and BbF, BkF, and Chry in dusts. Toxicity analysis revealed that PAHs with four rings, including carcinogenic PAHs, were the dominant pollutants in the studied area. The toxic equivalency value (TEQ_BaP_), the carcinogenic health risk assessment value recommended by the US EPA, was calculated for seven carcinogenic PAHs, revealing that they account for more than 93.0% of the total TEQ_Bap_ of the 16 PAHs and indicating the major toxic equivalent concentration contributor. Incremental lifetime cancer risk (ILCR) estimation results showed that PAHs tended to bring about great health risks through skin contact, followed by ingestion and inhalation. By comparison, road dust exhibited greater carcinogenic risks than roof dust, and bare soil may undergo heavier pollution. Therefore, the results of this study would be helpful in the effort to understand the PAHs pollution from the steel industry, which will provide some guidance for the probabilistic assessment of local health risks.

## Introduction

Polycyclic aromatic hydrocarbons (PAHs), a class of persistent semi-volatile organic pollutants with characteristics of high toxicity and strong metamorphism, are one of the first discovered environmental carcinogens^1–3^. PAHs consist of over 200 organic compounds, of which 16 are included in the list of priority controlled pollutants by the United States Environmental Protection Agency^4^. Numerous studies have revealed the main sources of PAHs to be residential coal burning, garbage incineration, activation of internal combustion engines, and various industrial activities such as coke production, oil refining, aluminum production, and smelting of non-ferrous metals^5^. It is reported that PAH pollution in industrial areas is more serious than that in residential areas; thus, many studies have reported PAH concentrations in the areas surrounding coal storage, coking and power plants, and iron and steel enterprises (ISE)^6–8^. Notably, the ISE performs multiple production steps and long-milling techniques, such as sintering, coking, iron smelting, and steelmaking, and each step contains several combined processes that lead to PAH pollution^9^.

With improvements in technology, particles and PAHs from tail pipe emissions have been significantly reduced. However, atmospheric PAHs, which escape photo-degradation in the air and treatment from the tail gas equipment can accumulate in environmental media through dry and wet deposition^10^. Moreover, with the expansion of industrialization and urbanization in China, PAH emissions have maintained an increasing trend, which means that the impact of PAHs on society has gradually increased^11^. It is reported that PAH levels have clear temporal and spatial distribution characteristics in China: PAH concentrations in winter are significantly higher than in other seasons^12,13^, and in northern cities, they are higher than in southern cities^14^. Researchers have given attention to the characteristics, concentrations, and sources of PAHs in different environmental media^15,16^. In most of these studies, the status of PAH pollution has been evaluated in the surrounding soil, street dust, water, atmosphere, and other environmental media or biological systems^17–20^, whereas research on PAHs in different types of dust is quite limited. In addition, it is very important to study the influence of PAHs on the surrounding environment in air-dust media.

Laiwu lies in the middle of Shandong Province that the third largest economic province in northern China and has iron and steel production plants. It has an annual production capacity of 3 million tons of fine metal plates, sheets, and strips and 600,000 tons of stainless steel. In the present study, road dust, roof dust, bare soil, and atmosphere particles were collected from the ISE. The concentration level and environmental impact of 16 PAHs in dusts surrounding the ISE were assessed. Also, incremental lifetime carcinogenic risk due to exposure to PAHs in dust was evaluated. These findings can serve as a scientific basis for the control of PAH pollution in the areas surrounding the ISE.

## Materials and methods

### Sample sites and collection

According to the main producing process of ISE, dominant wind direction of the region and the surrounding villages, five sample sites surrounding an ISE were selected based on their representativeness of the area in Laiwu City. The detailed information for the sampling sites are as follows: site 1 (S1): coking plant, site 2 (S2): iron smelting plant (including sintering), site 3 (S3): steelmaking plant: site 4 (S4): Daqin Village (located 3.6 km northeast of the nearest ISE boundary), site 5 (S5): Mengjiazhuang (located 2.2 km southwest of the nearest ISE boundary). The dominant wind direction surrounding the ISE is greatly controlled by the northeast and southeast wind in winter (our sampling period).

**Figure 1:**
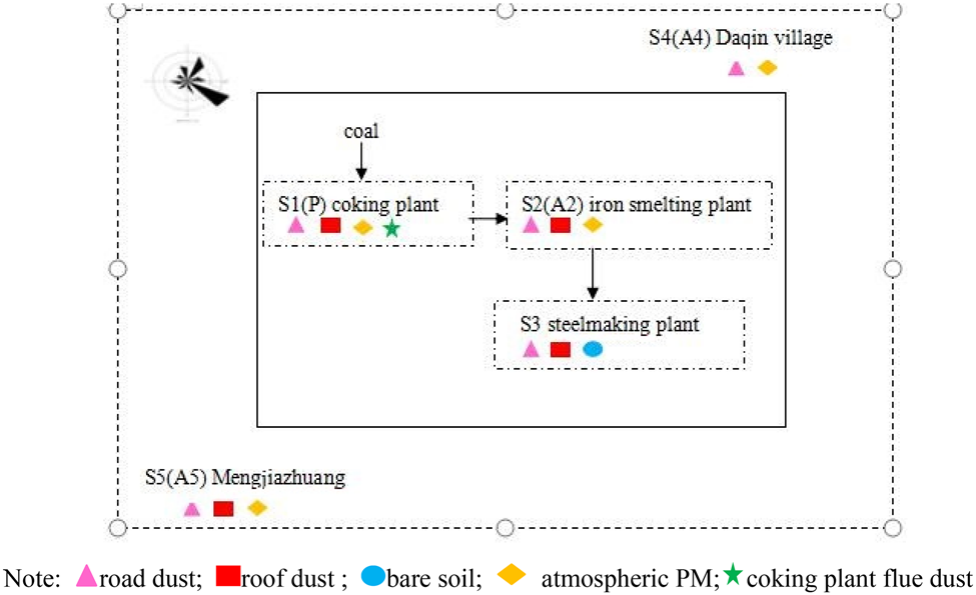
Spatial distribution of the sampling sites.

The spatial distribution of the sampling sites is shown in Fig. 1. Road dust samples were collected at five sites (S1–S5). Roof dust samples were collected at S1, S2, S3, and S5, at height of approximately 10 m, 10 m, 10 m, and 15 m above the ground, respectively. The atmospheric particulate matter (PM) samples (A2, A4, A5) were collected at S2, S4, and S5, at heights of approximately 10 m, 13 m, and 15 m above the ground, respectively. Moreover, bare soils were collected at S3. For each type of sample, at least three samples were collected at the same site. Dust particle samples (A1) emitted from the the ISE coking plant were collected at S1.

Road and roof dust samples were collected about 150 g using vacuum cleaners (Samsung, SC88P0). Bare soils were collected up about 500 g to a depth of 20 cm at S3 by shovel. The collected raw road, roof dust, and bare soil samples were air-dried indoors for a period till to the moisture content nearly zero, and then sieved through a 48 mesh sieve (equal to 300 μm) to pretreat the raw dust samples(the screen underflow sample was named laser analysing sample) and then select about 50 g laser analysing sample use 150 mesh sieve (equal to 100 μm) to retreat the samples, and kept the screen underflow for further PAHs analysis(the screen underflow sample was named PAHs analysing sample). Atmospheric PM filter samples (PM_2.5_ and PM_10_) were collected at A2, A4, and A5 in winter (December 25–30, 2016). Each quartz filter sample (φ90 mm) of PM with aerodynamic diameters ≤ 2.5 μm (PM_2.5_) and aerodynamic diameters ≤ 10 μm (PM_10_) was collected for 24 h using a median-flow particle sampler (Tianhong, Wuhan, Co. Ltd) with a flow rate of 100 L/min. The dust quartz filter sample (P) from the coking plant chimney at S1 was collected for 20 h to one sample with a flow rate 16.67 L/min by diluting channel sampling equipment (made by Qingdao Laoshan Ltd., Qingdao, China), which was calibrated by a gas mass flow calibrator (API 700, New York, NY, USA).

### Sample pretreatment and instrumental analysis

Approximately 1.0 g of each PAHs analysing sample (including road, roof dust samples and bare soils) and quartz filter sample (including the atmospheric PM Quartz filter samples and one dust particle sample from the coking plant chimney), were extracted for 16 h at 60 °C with n-hexane, using a set of soxhlet extractors, respectively. The extractant was concentrated to 2–3 mL by a rotary evaporator and then purified through a silica gel column. Then, the eluents were collected and concentrated to 0.5 mL, followed by dilution to 1 mL with n-hexane for the subsequent analysis.

The analysis of PAHs was performed with an electrospray ionization source in single reaction monitoring mode using gas chromatography mass spectrometry with a DB-5MS column (60 m × 0.25 mm × 0.25 μm; Agilent). The instrumental analysis conditions were set as the following: injection temperature of 250 °C, column flow velocity of 1.10 mL/min, split flow ratio of 10:1; column pressure of 69.3 kPa, oven temperature of 40 °C, and sample quantity of 1.0 μL.

The raising temperature program was listed as follows: initial temperature of 70 °C for 1 min; warming to 240 °C at a heating rate of 20 °C/min; and warming to 310 °C at a heating rate of 10 °C/min and maintained for 20 min. The carrier gas was high-purity nitrogen.

The target compounds for monitoring and analysis were 16 types of US EPA PAHs, and the specific substances and their properties, limit of detection, and limit of quantification were shown in Table 1.

**Table 1:** Names and properties of 16 US EPA priority PAHs.

Serial number	Name	Abbreviations	Number of benzene rings	Limit of detection (μg/mL)	Limit of quantification (μg/mL)
1	Naphthalene	NaP	2	0.002	0.005
2	Acenaphthene	Ace	3	0.004	0.01
3	Acenaphthylene	Acy	3	0.005	0.012
4	Fluorine	Flu	3	0.005	0.013
5	Phenanthrene	Phe	3	0.003	0.008
6	Anthracene	Ant	3	0.004	0.011
7	Fluoranthene	Flua	4	0.007	0.124
8	Pyrene	Pyr	4	0.004	0.013
9	Benzo[a]anthracene	BaA	4	0.002	0.007
10	Chrysene	Chry	4	0.007	0.013
11	Benzo[*b*]fluoranthene	BbF	5	0.003	0.006
12	Benzo[*k*]fluoranthene fluoranthene	BkF	5	0.004	0.013
13	Benzo[a]pyrene	BaP	5	0.004	0.007
14	Indeno [1,2,3-*cd*] pyrene	IcdP	6	0.005	0.015
15	Dibenz[ah]anthracene	DahA	5	0.006	0.013
16	Benzo[ghi]perylene	BghiP	6	0.004	0.013

### Quality control

Among the 16 types of PAHs, only BaA, Chry, IcdP, and DahA of 16 PAHs were detected in the analytical blank samples, whereas others could not be detected within their limit of detection. The detected blank samples average concentration of BaA, Chry, IcdP, and DahA, was 0.0031, 0.0085, 0.0055, and 0.0067 μg/mL, which was very low, indicating that little interference for the target compounds was present in the experiment. These blank samples concentrations were subtracted from the concentrations in the actual samples to account for the blank contamination. The 16 types of PAHs mixed standard solutions (2000 μg/mL, AccuStandard Inc., US) with concentration of 0.2, 0.4, 0.6, 0.8, 1.0, and 2.0 mg/L were configured, with correlation coefficients, R^2^, all above 0.9997. For every ten samples indicator perylen-d12 standard solution (4000 µg/mL, AccuStandard Inc., US) was added to one actual sample and blank sample for the QA/QC, and the recovery efficiency was between 82 and 113% and 78 and 100%, respectively (meeting the EPA requirement 80–120%), and the relative standard deviation (RSD) was 1.74–12.6% and 3.2–11.5%, respectively (meeting the EPA requirement of RSD < 20%).

### Particle size analysis method

The laser particle size analyzer (LS-C(I), Zhuhai Omec Company, Zhuhai, China) was used to test the particle size distribution of two typical samples of road dust and roof dust. Before testing the size distribution, the raw dust particle samples were pretreated by 300 µm stainless steel sieve and the pretreated method was listed in 1.1 (laser analyzing sample).

### Risk assessment methods

The toxic equivalency value of BaP (TEQ_BaP_) is used to evaluate the potential ecological risk caused by the PAHs^21^. The calculation method is given in Eq. (1).

1 $$ {\text{TEQ}}_{{{\text{Bap}}}} = \sum {({\text{C}}_{{\text{i}}} \times {\text{TEF}}_{{\text{i}}} )} , $$

where C_i_ is the concentration of the ith type of PAHs μg/g), TEF_i_ is the toxic equivalency factor of the ith type of PAHs (Table 3), and TEQ_BaP_ is the BaP-based toxic equivalency value (µg/g)^22^.

The carcinogenic risk of PAHs to human health is manifested in three ways: direct ingestion, inhalation, and dermal contact^23^. The effect of PAHs on human health is calculated according to the carcinogenic health risk assessment model recommended by the US EPA^24^. The calculation formulas are listed as follows.

2 $$ {\text{ILCR}}_{{{\text{ing}}}} = \frac{{{\text{TEQ}}_{{{\text{Bap}}}} \times {\text{CSF}}_{{{\text{ing}}}} \times \sqrt[3]{{{\text{BW}}/70}} \times {\text{IR}}_{{{\text{ing}}}} \times {\text{EF}} \times {\text{ED}}}}{{{\text{BW}} \times {\text{AT}} \times 10^{6} }}, $$

3 $$ {\text{ILCR}}_{{{\text{inh}}}} = \frac{{{\text{TEQ}}_{{{\text{Bap}}}} \times {\text{CSF}}_{{{\text{inh}}}} \times \sqrt[3]{{{\text{BW}}/70}} \times {\text{IR}}_{{{\text{inh}}}} \times {\text{EF}} \times {\text{ED}}}}{{{\text{BW}} \times {\text{AT}} \times {\text{PEF}}}}, $$

4 $$ {\text{ILCR}}_{{{\text{derm}}}} = \frac{{{\text{TEQ}}_{{{\text{Bap}}}} \times {\text{CSF}}_{{{\text{derm}}}} \times \sqrt[3]{{{\text{BW/}}70}} \times {\text{SA}} \times {\text{SL}} \times {\text{ABS}} \times {\text{EF}} \times {\text{ED}}}}{{{\text{BW}} \times {\text{AT}} \times 10^{6} }}, $$

5 $$ {\text{TILCR}} = {\text{ILCR}}_{{{\text{ing}}}} + {\text{ILCR}}_{{{\text{inh}}}} + {\text{ILCR}}_{{{\text{derm}}}} . $$

In Eqs. (2–5), ILCR_ing_, ILCR_inh_, and ILCDR_derm_ are the carcinogenic health risk values of ingestion, inhalation, and skin contact, respectively. TILCR is the sum of the three carcinogenic health risks; IR_ing_ is the ingesting rate (mg/day); IR_inh_ is the inhalation rate (m^3^/day), EF is the exposure frequency (d/a), ED is the exposure duration (a), BW is the body weight (kg), AT is the average exposure time (a), PEF is the particulate matter emission factor (m^3^/kg); SL is the skin adhesion [mg/(cm^2^ day)], SA is the exposed skin area (cm^2^), ABS is the skin absorption factor; and CSF_ing_, CSF_inh_, and CSF_derm_ are the carcinogenic slope coefficients of the three exposure pathways, which are 7.3, 3.85, and 25.0, respectively (kg d)/mg. When ILCR or TILCR is below 10^–6^, between 10^–6^ and 10^–4^, or above 10^–4^, this means that there is no carcinogenic risk, low to moderate carcinogenic risk, or high carcinogenic risk, respectively^25^.

## Results and discussion

16 US EPA priority PAH concentrations in road dust (RD), roof dust (RF), and bare soil (BS) at different sites were shown in Table 2.

**Table 2:** Concentration of PAHs (μg/g) in the RD, RF, and BS from five sites surrounding the ISE.

PAHs	S1		S2		S3		S4		S5	
	RD	RF	RD	RF	BS	RD	RF	RD	RD	RF
NaP	0.730 ± 0.080	0.970 ± 0.092	0.160 ± 0.014	0.080 ± 0.071	0.990 ± 0.088	0.020 ± 0.018	0.070 ± 0.008	N.D	0.060 ± 0.053	0.020 ± 0.002
Acy	1.610 ± 0.153	0.260 ± 0.025	0.02 ± 0.002	0.010 ± 0.001	0.040 ± 0.004	0.020 ± 0.002	0.010 ± 0.001	N.D	0.010 ± 0.001	N.D
Ace	0.140 ± 0.093	0.090 ± 0.008	0.02 ± 0.002	0.010 ± 0.001	0.100 ± 0.009	0.010 ± 0.001	0.010 ± 0.001	N.D	0.010 ± 0.001	0.010 ± 0.001
Flu	0.730 ± 0.069	0.420 ± 0.040	0.09 ± 0.011	0.030 ± 0.027	0.150 ± 0.015	0.010 ± 0.001	0.010 ± 0.001	N.D	0.050 ± 0.005	N.D
Phe	3.570 ± 0.303	1.700 ± 0.162	0.21 ± 0.019	0.250 ± 0.024	1.070 ± 0.102	0.120 ± 0.012	0.230 ± 0.025	0.020 ± 0.002	0.290 ± 0.028	0.060 ± 0.007
Ant	1.440 ± 0.130	0.350 ± 0.040	0.040 ± 0.004	0.030 ± 0.004	0.080 ± 0.010	0.040 ± 0.005	0.020 ± 0.002	0.010 ± 0.001	0.050 ± 0.006	0.010 ± 0.001
Flua	4.620 ± 0.439	1.970 ± 0.213	0.130 ± 0.014	0.360 ± 0.032	1.310 ± 0.144	0.260 ± 0.025	0.470 ± 0.042	0.040 ± 0.004	0.260 ± 0.023	0.060 ± 0.006
Pyr	3.060 ± 0.127	1.250 ± 0.113	0.120 ± 0.012	0.270 ± 0.020	1.130 ± 0.021	0.160 ± 0.014	0.260 ± 0.029	0.030 ± 0.004	0.330 ± 0.036	0.040 ± 0.005
BaA	3.350 ± 0.285	0.990 ± 0.089	0.160 ± 0.018	1.730 ± 0.156	0.540 ± 0.049	0.180 ± 0.015	0.430 ± 0.041	0.050 ± 0.005	0.590 ± 0.053	0.080 ± 0.007
Chry	3.430 ± 0.307	1.820 ± 0.127	0.170 ± 0.015	1.900 ± 0.171	2.710 ± 0.300	0.330 ± 0.031	0.460 ± 0.044	0.050 ± 0.006	0.640 ± 0.061	0.080 ± 0.010
BbF	7.270 ± 0.118	2.280 ± 0.196	0.070 ± 0.008	0.580 ± 0.055	2.140 ± 0.311	0.360 ± 0.034	0.240 ± 0.023	0.060 ± 0.053	0.290 ± 0.025	0.070 ± 0.008
BkF	7.270 ± 0.121	2.280 ± 0.196	0.070 ± 0.008	0.580 ± 0.055	1.820 ± 0.164	0.360 ± 0.020	0.240 ± 0.021	0.060 ± 0.005	0.290 ± 0.021	0.070 ± 0.008
BaP	3.030 ± 0.118	0.880 ± 0.079	0.030 ± 0.003	0.210 ± 0.021	0.630 ± 0.069	0.120 ± 0.013	0.070 ± 0.006	0.040 ± 0.004	0.210 ± 0.019	0.040 ± 0.004
IcdP	2.730 ± 0.127	0.710 ± 0.067	0.030 ± 0.004	0.160 ± 0.017	0.410 ± 0.044	0.080 ± 0.071	0.050 ± 0.006	0.040 ± 0.004	0.120 ± 0.013	0.050 ± 0.007
DahA	1.190 ± 0.107	0.280 ± 0.025	0.030 ± 0.003	0.100 ± 0.009	0.170 ± 0.015	0.050 ± 0.044	0.030 ± 0.027	0.030 ± 0.002	0.100 ± 0.013	0.030 ± 0.004
BghiP	2.800 ± 0.122	0.890 ± 0.080	0.030 ± 0.003	0.260 ± 0.023	0.700 ± 0.067	0.090 ± 0.080	0.060 ± 0.007	0.040 ± 0.004	0.210 ± 0.186	0.050 ± 0.005
∑_16_ PAHs	46.970 ± 4.791	17.140 ± 4.462	1.370 ± 0.117	6.550 ± 0.583	13.990 ± 1.203	2.210 ± 0.188	2.660 ± 0.246	0.460 ± 0.043	3.510 ± 0.325	0.670 ± 0.062

*N.D.* not detected.

### Concentration level of PAHs in dusts

#### Concentration level of PAHs in road dust

The total concentration of the 16 PAHs (∑_16_ PAHs) in the road dust samples ranged from (0.460 ± 0.043) to (46.970 ± 4.791) μg/g in Table 2, with an average value of (10.892 ± 1.185) μg/g. Among the five sampling sites, the highest ∑_16_ PAHs value was found at S1, followed by S5 and S3, and the lowest values appeared at S4, upwind of the ISE. The S1 sampling site, located near the coking plant, had the highest PAH concentration level. This phenomenon was consistent with conclusions from previous studies that showed that the sedimentation rate of PAHs near iron and steel works was much greater than that of the other zones^26^. Moreover, the wind direction can significantly affect the PAH concentrations. The ∑_16_ PAHs concentration at S5, downwind of the ISE, was (3.510 ± 0.325) µg/g and was approximately 7 times the value at S4, upwind of the ISE. In addition, the individual PAH concentrations were all below 0.1 µg/g, suggesting that it can be affected by wind direction. Vasilakos et al.^27^ reported that both wind speed and wind direction had an effect on the PAHs concentrations with the source of the pollution coming from different directions.

The most abundant individual PAHs were BbF and BkF in the road dust samples at S1, S3, and S4. However, the dominant compounds were Phe and Chry at S2 and Chry and BaA at S5. Carcinogenic BaP was detected in all dust samples, and the BaP concentration at site S1 near the coking plant had a higher value than that at other sites. Comparison with other reports shows that the average concentrations of PAHs in road dust near the ISE were similar, with a value of 10.62 μg/g in Xi’an^28^, slightly higher than that in Shanghai and Sydney^29,30^. Notably, in our study area, only the PAHs concentration level at S1 was much higher than previously reported levels, and the concentrations at the other sampling sites were far below those reported in the literature.

#### Concentration level of PAHs in roof dust

In Table 2, the total concentration of the 16 PAHs was between (0.670 ± 0.062) μg/g and (17.140 ± 4.462) μg/g in roof dust, with an average value of (6.751 ± 0.692) μg/g. The highest ∑_16_PAHs value was found at S1, followed by S2 (6.550 ± 0.583 µg/g) and S3 (2.660 ± 0.246 μg/g), and the lowest values appeared at S5. Because the S1 sampling site was located near the coking plant, the roof dust at this site had the highest PAH concentration. This phenomenon was in accordance with the level of ∑_16_ PAHs in road dust at the S1 site (Table 2). As is known, PAHs are more readily generated during the incomplete combustion of fossil fuels in the coking process, especially in the absence of oxygen^31^. Moreover, the PAH with the highest concentration of roof dust differed at four sites, in which BbF and BkF, Chry, Flua, and BaA and Chry exhibited the highest roof dust concentrations at S1, S2, S3, and S5, respectively. Interestingly, the most abundant PAH for roof dust and road dust at S1 were similar. There is little published data regarding the concentration of PAHs in roof dust; thus, our results are significant in providing some guidance on PAHs pollution.

#### Distribution of PAHs in dust with sampling height

**Figure 2:**
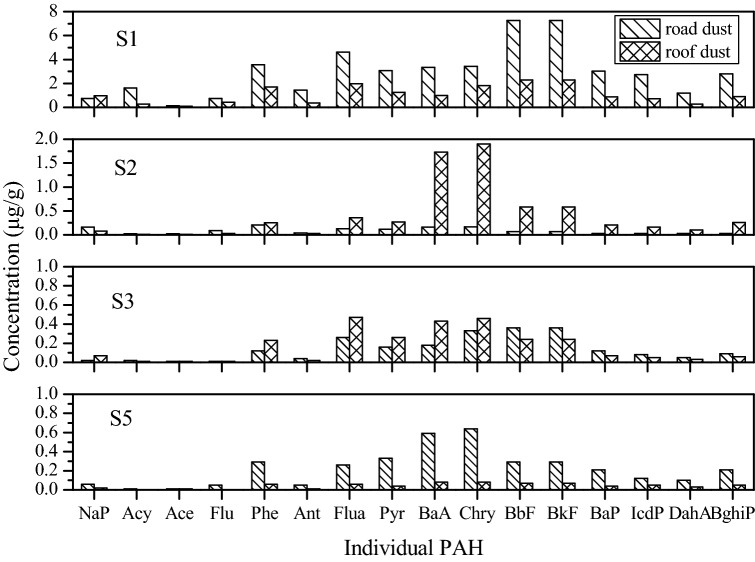
Distribution of PAH of road dust and roof dust at various sampling sites.

Road dust and roof dust at the same sample sites were collected from different heights, representing the surface ground (low height) and 10 m height above ground (high height). The distribution of PAHs in dust at different sampling heights was shown in Fig. 2. The PAHs concentrations were higher in roof dust than in road dust at S2 and S3, whereas they were higher in road dust at S1 and S5. The ∑_16_ PAHs level of road dust was almost 2.74 and 5.24 times higher than that of roof dust at S1 and S5, respectively; for example, BbF and BkF at S1 and BaA and Chry at S5 were significantly higher in road dust than in roof dust. Meanwhile, the ∑_16_ PAHs concentration of road dust was 0.21 and 0.82 times lower than that of roof dust at S2 and S3, respectively; for instance, BaA and Chry at S2 were remarkably higher in roof dust than in road dust. This is mainly because the combustion of coal in the coking plant at S1 was a dry distillation process; meanwhile, severe hypoxia and high temperatures in the furnace were conducive to the generation of PAHs. The dust on the roof is not only the receiver of PAHs discharged from the coking plant flue but also the secondary contributors to the road dust. Because of its complex sources, the order of PAH concentrations for the road dust and roof dust was different at the four sampling sites, mainly attributed to the influence of the different sampling sites and the distance to the different industrial processes of the ISE.

**Figure 3:**
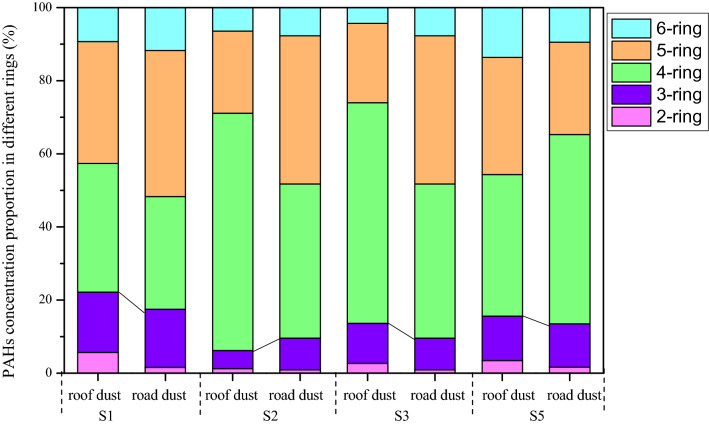
Distribution of PAH with various rings at the sampling sites.

PAHs containing four or more rings are defined as high-ring PAHs, whereas others are considered to be low-ring PAHs^32^. The ring distribution of PAHs in the dust samples are presented in Fig. 3. In this study, different PAH sources, such as road dust and roof dust, have a similar PAH ring distribution such that high-ring PAHs had a higher proportion than low-ring PAHs. Four-ring PAH concentrations were the highest in roof dust at S2 and S3, and road dust at S5, whereas PAHs with four and five rings were both abundant at the other sites, followed by PAHs with three rings. Moreover, high-ring PAHs were mainly present on the surface of the road dust, whereas low-ring PAHs were more likely to exist on roof dust. The abundance of three-to-five-ring PAHs in the dust indicated that the dusts were exposed for a long time to PAHs that originated from industrial activities in these areas.

**Figure 4:**
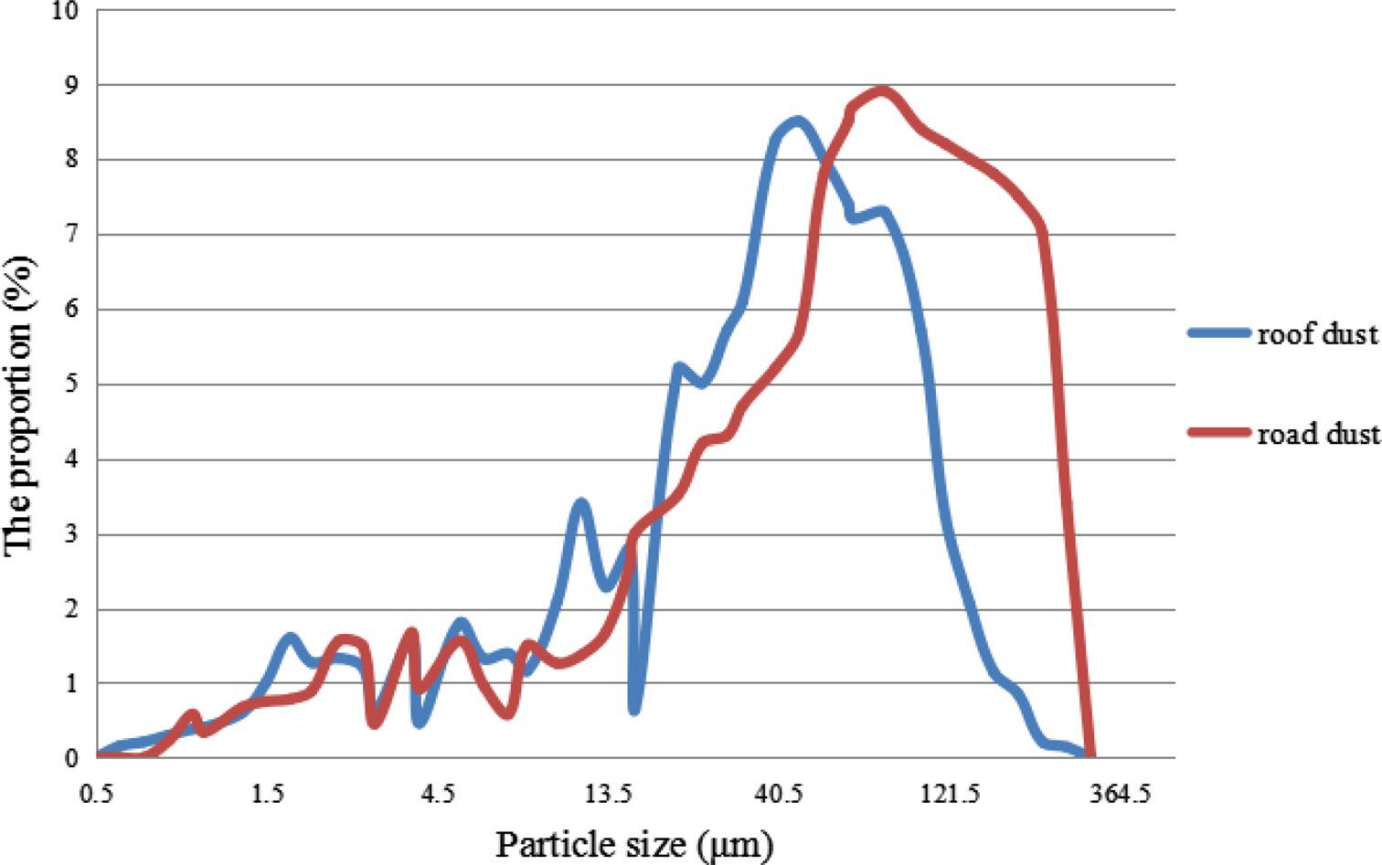
Distribution of particle size in roof dust and road dust.

Typical samples of roof dust and road dust were collected to illustrate their particle size distribution. As were shown in Fig. 4, road dust sample showed a skewed distribution and leaned toward larger particles, whereas roof dust sample exhibited a normal distribution. The peak of the particle size distribution of roof dust and road dust reached 46.13 μm and 80.46 μm, in which the maximum proportion was 8.50% and 8.91%, respectively. D_10_–D_90_ in road dust (12.42–200.32 μm) was larger than that in roof dust (5.44–149.16 μm), which indicated the compositional complexity of road dust. The D_50_ of road dust (75.30 μm) was higher than that of roof dust (32.63 μm). The particle size of roof dust was finer, whereas the particle size of road dust was coarser.

#### Concentration level of PAHs in bare soil

The sampling site of bare soil in this study is located in the steelmaking plant (S3). The total concentration of PAHs (Ʃ_16_ PAHs) was 13.990 ± 1.203 μg/g in bare soil (Table 2). Among these PAHs, Chry had the highest concentration (2.710 ± 0.300 μg/g), followed by BbF (2.140 ± 0.311 µg/g) and BkF (1.820 ± 0.164 μg/g). The concentrations of Acy, Ant, Ace, and Flu were quite lower. Notably, the concentration of BaP, a carcinogenic PAH, was 0.630 ± 0.069 μg/g.

Currently, some reports are available concerning the concentration of PAHs in the soil surrounding steelmaking plants in China. Dong et al.^33^ found the concentrations of the sixteen PAHs to be between 0.02 and 20.06 μg/g (mean value of 2.56 μg/g), among which the BaP concentration was 0.16 μg/g in soil surrounding a steelmaking plant in northern China. However, Tian et al.^34^ reported a higher concentration of Ʃ_16_PAHs and BaP of 32.10 and 0.58 μg/g, respectively, at another steelmaking plant located in northeastern China. Furthermore, the total concentration of these PAHs was up to 32.45 μg/g at a coking plant in Beijing^35^. From these results, we found the concentration of PAHs of bare soil in our study to be near an average level; it was below average around the steelmaking and coking plants in northeastern China and Beijing but above average near the steelmaking plant in northern China.

Chry is one of the carcinogenic PAHs from coal combustion^36,37^. The Chry concentration was up to 2.710 ± 0.300 μg/g in our study, which was much higher than the previous reports of 1.57 μg/g^38^. There is no currently published evaluation standard for soil PAHs in China; thus, the Canadian soil quality benchmark was used to assess soil quality^39^. The concentration of BaP was 0.630 ± 0.069 μg/g in the collected bare soil, which was higher than the reference value of 0.10 μg/g in agricultural soil. Thus, it was indicative of severe pollution in the bare soils in the tested regions.

In summary, different sampling areas contained different PAHs; however, the main types of PAHs in road dust, roof dust, and bare soil at the same sampling site were similar: BbF and BkF were the main pollutants in road and roof dust at S1; BaP and Chry were the main pollutants at S2; the main pollutants were Chry, BbF, and BkF in the bare soil, road dust, and roof dust at S3; the main pollutants were BbF and BkF from road dust at S4; and at S5, the main PAHs were BaP and Chry.

### Impact of PAHs in dust on environment atmospheric PM

**Figure 5:**
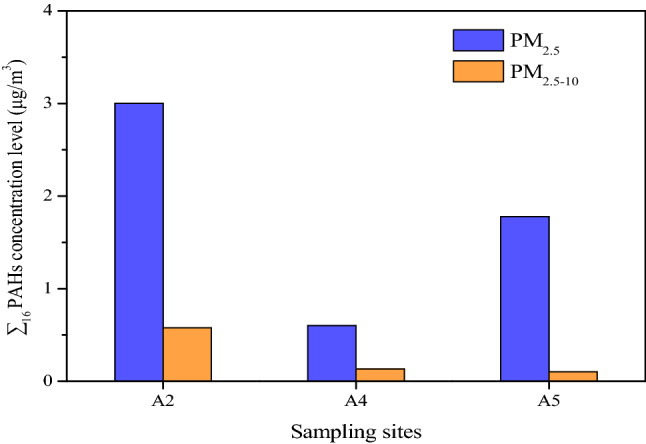
∑_16_ PAHs concentrations in atmospheric particulate matter (PM), roof dust, and road dust at different sampling sites.

The ∑_16_ PAHs concentration in PM_2.5_ and PM_2.5–10_ at atmospheric PM sites (A2, A4, A5) were shown in Fig. 5. The ∑_16_ PAHs concentration was the highest at A2 inside the iron-smelting plant of the ISE, at 3.01 µg/m^3^ in PM_2.5_ and 0.58 µg/m^3^ in PM_2.5–10_, respectively, and the concentration of ∑_16_ PAHs in A4, upwind of the ISE, was remarkably lower than that in A5, downwind of the ISE. This confirmed that the ISE had a heavy environmental impact on PAHs in the surrounding atmospheric PM, consistent with the research conclusions of related reference^40^. The mass concentration of ∑_16_ PAHs in PM_2.5_ was 9.741 µg/m^3^ in the environmental air, approximately 1 km from a coking plant^41^. The ∑_16_ PAHs concentration in our collected samples was lower than that in the literature. The ∑_16_ PAHs concentration proportion of PM_2.5_ was 81.83% (A2), 83.84% (A4), and 94.49% (A5) of that in PM_10_. Such a high proportion suggested that PAHs of finer particle size should be given significant emphasis.

**Figure 6:**
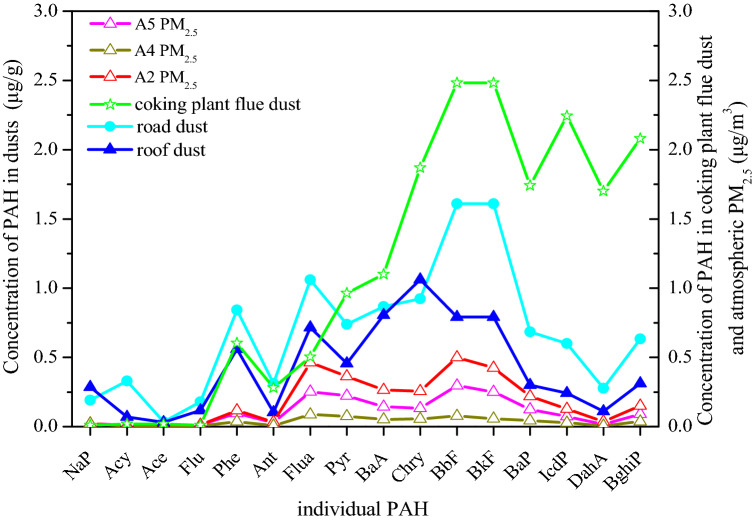
Individual PAH concentrations in dust, coking plant, flue dust, and atmospheric PM_2.5_.

To reflect the environment impact of individual PAHs from dusts on atmospheric PM, flue dust from one coking plant was collected to compare with the individual PAHs of roof dust and road dust by averaging the data from the different sites. As shown in Fig. 6, road dust and roof dust exhibit similar concentration curves, in which BbF and BkF were the main components of roof dust and road dust, and the PAH with the highest concentration in road dust was Chry. BbF and BkF were also the dominant individual PAHs of the flue dust, whereas the concentration of high-ring PAHs, including BaP, IcdP, DahA, and Behia, was also high. This could be explained as the PAHs with more rings underwent complex physical and chemical reactions in the atmosphere after being discharged from the flue dust, causing a transformation into other environment media. It is reported that high-ring PAHs were derived from the incomplete combustion of fossil fuels and more likely to be adsorbed onto the soil and dust particles^6,42^. This might also explain the distribution of PAHs in the present study. For atmospheric PM_2.5_, the concentration curves of the different sample sites were similar; the concentration of BbF was the highest, followed by Flua and BkF. Moreover, the concentration of BaP, a heavily carcinogenic PAH, was 0.50, 0.08, and 0.30 μg/m^3^ in A2, A4, and A5, respectively, all exceeding the 2nd standard value of the National Ambient Air Quality Standard of BaP (0.0025 µg/m^3^) in China. Furthermore, BaP/∑_16_ PAHs ranged from 7.03 to 9.62% in atmospheric PM_2.5_ and from 4.44 to 6.28% in dusts. Hence, the higher BaP concentration and BaP/∑_16_ PAHs value suggested that atmospheric PAHs should be paid much more attention, as they may have a serious impact on the surrounding atmosphere.

### Probabilistic health risk assessment of PAHs

The toxic equivalent factors and equivalency values and the carcinogenic risk evaluation results of the 16 PAHs were listed in Table 3. The BaP-based toxic equivalency (TEQ_BaP_) values exhibited great differences, depending on the sampling site. For example, the TEQ_BaP_ of the sixteen PAHs was the highest at S1, (1.823 ± 0.091)–(6.370 ± 0.306) μg/g, and it was (0.095 ± 0.008)–(0.638 ± 0.031) μg/g, (0.202 ± 0.017)–(1.330 ± 0.064) μg/g, 0.085 ± 0.004 μg/g, and (0.986 ± 0.009)–(0.443 ± 0.021) μg/g at S2, S3, S4, and S5, respectively (Table 2). The TEQ_BaP_ of the seven carcinogenic PAHs accounted for (93.760 ± 7.969)–(95.590 ± 8.125)% of the total TEQ_Bap_ of 16 PAHs, indicating that these carcinogenic PAHs led to the ecological risk. Among the seven carcinogenic PAHs, the health risk of BaP was the highest. The Σ_16_TEQ_BaP_ of the various dust samples followed the order: road dust at S1 (6.370 ± 0.306 μg/g) > roof dust at S1 (1.823 ± 0.091 μg/g) > bare soil at S3 (1.330 ± 0.064 μg/g) > roof dust at S2 (0.638 ± 0.031 μg/g) > road dust at S5 (0.443 ± 0.021 μg/g) > road dust at S3 (0.272 ± 0.013 μg/g) > roof dust at S4 (0.202 ± 0.017 μg/g) > roof dust at S5 (0.099 ± 0.009 μg/g) > road dust at S2 (0.096 ± 0.008 μg/g) > road dust at S4 (0.085 ± 0.004 μg/g). Evidently, S1 demonstrated the highest Σ_16_TEQ_BaP_. Among the various dust samples, the Σ_16_TEQ_BaP_ values were higher in road dust than that in roof dust, with the exception of that at S2. There are few reports on the TEQs of PAHs in dust samples. Taking that into consideration, a rough comparison was made regarding the TEQs of PAHs in soils from the different sampling sites in and around an ISE, which showed that the Σ_16_TEQ_BaP_ and Σ_7_TEQ_BaP_ were 0.340 and 0.330 μg/g, respectively, and the Σ_7_TEQ_BaP_ to Σ_16_TEQ_BaP_ ranged from 76.400 to 99.100%^43^. These findings indicated that carcinogenic PAHs were the main contributors to the total TEQ_BaP_. Moreover, the concentration of carcinogenic PAHs in our study was higher than that Tao reported in the literature^43^, suggesting a heavier potential ecological risk for these carcinogenic PAHs in the investigated regions.

**Table 3:** Health risk caused by PAHs in RD, RF, and BS inside the ISE and in the surrounding environment based on the TEQ_BaP_ (× 10^–3^ μg/g).

PAH	TEF	S1		S2		S3		S4		S5	
		Rd	Rf	Rd	Rf	Bs	Rd	Rf	Rd	Rd	Rf
NaP	0.001	0.728 ± 0.061	0.970 ± 0.077	0.155 ± 0.017	0.080 ± 0.010	0.992 ± 0.011	0.020 ± 0.002	0.070 ± 0.008	N.D	0.057 ± 0.007	0.020 ± 0.002
Acy	0.001	1.611 ± 0.128	0.260 ± 0.020	0.016 ± 0.001	0.010 ± 0.010	0.043 ± 0.046	0.016 ± 0.002	0.010 ± 0.001	N.D	0.010 ± 0.001	N.D
Ace	0.001	0.136 ± 0.010	0.090 ± 0.010	0.023 ± 0.002	0.010 ± 0.197	0.103 ± 0.185	0.006 ± 0.001	0.010 ± 0.001	N.D	0.012 ± 0.001	0.010 ± 0.001
Flu	0.001	0.731 ± 0.057	0.420 ± 0.033	0.094 ± 0.007	0.030 ± 0.406	0.148 ± 0.082	0.013 ± 0.001	0.010 ± 0.001	N.D	0.054 ± 0.006	N.D
Phe	0.001	3.573 ± 0.278	1.700 ± 0.144	0.208 ± 0.016	0.250 ± 0.026	1.073 ± 0.091	0.120 ± 0.014	0.230 ± 0.018	0.024 ± 0.003	0.290 ± 0.025	0.060 ± 0.006
Ant	0.01	14.350 ± 1.119	3.500 ± 0.273	0.380 ± 0.030	0.300 ± 0.026	0.750 ± 0.064	0.350 ± 0.030	0.200 ± 0.017	0.070 ± 0.008	0.510 ± 0.043	0.100 ± 0.010
Flua	0.001	4.618 ± 0.360	1.970 ± 0.167	0.130 ± 0.011	0.360 ± 0.031	1.307 ± 0.111	0.261 ± 0.022	0.470 ± 0.040	0.035 ± 0.004	0.260 ± 0.022	0.060 ± 0.006
Pyr	0.001	3.064 ± 0.238	1.250 ± 0.106	0.115 ± 0.009	0.270 ± 0.023	1.128 ± 0.096	0.158 ± 0.013	0.260 ± 0.022	0.032 ± 0.004	0.326 ± 0.028	0.040 ± 0.004
BaA*	0.1	334.700 ± 24.098	99.000 ± 7.722	16.300 ± 1.271	173.000 ± 13.494	53.460 ± 4.170	18.300 ± 1.427	43.000 ± 3.354	5.100 ± 0.434	59.100 ± 4.610	8.000 ± 0.816
Chry*	0.01	34.330 ± 2.677	18.200 ± 0.873	1.680 ± 0.131	19.000 ± 1.520	27.107 ± 2.006	3.250 ± 0.241	4.600 ± 0.340	0.510 ± 0.043	6.400 ± 0.544	0.800 ± 0.082
BbF*	0.1	727.300 ± 52.365	228.000 ± 17.432	6.500 ± 0.507	58.000 ± 4.524	214.17 ± 16.705	36.200 ± 2.824	24.000 ± 2.864	5.700 ± 0.422	29.200 ± 2.278	7.000 ± 0.595
BkF*	0.1	727.300 ± 53.820	228.00 ± 17.784	6.500 ± 0.533	58.000 ± 4.526	181.515 ± 14.158	36.200 ± 2.823	24.000 ± 1.872	5.700 ± 0.445	29.200 ± 1.119	7.000 ± 0.574
BaP*	1	3026.000 ± 217.872	880.000 ± 63.361	33.000 ± 2.574	210.000 ± 16.382	631.500 ± 46.731	121.000 ± 9.438	70.000 ± 5.460	37.000 ± 2.886	207.000 ± 14.904	40.000 ± 3.120
IcdP*	0.1	272.600 ± 21.262	71.000 ± 4.899	3.300 ± 0.227	16.000 ± 1.248	41.445 ± 3.233	8.300 ± 0.647	5.000 ± 0.425	3.800 ± 0.262	11.900 ± 0.928	5.000 ± 0.370
DahA*	1	1191.000 ± 88.134	280.000 ± 20.162	27.000 ± 2.106	100.000 ± 6.900	169.200 ± 13.198	47.000 ± 3.666	30.000 ± 2.340	27.000 ± 2.106	97.000 ± 7.566	30.000 ± 2.340
BghiP	0.01	28.000 ± 1.932	8.900 ± 0.614	0.340 ± 0.039	2.600 ± 0.299	6.948 ± 0.514	0.870 ± 0.102	0.600 ± 0.069	0.380 ± 0.044	2.130 ± 0.245	0.500 ± 0.058
∑_16_ TEQ_BaP_	/	6370.041 ± 305.761	1823.260 ± 91.163	95.741 ± 8.329	637.910 ± 30.621	1330.887 ± 63.883	272.06 ± 13.059	202.460 ± 17.209	85.351 ± 4.097	443.449 ± 21.286	98.590 ± 8.577
∑_7_ TEQ_BaP_	/	6038.920 ± 271.751	1725.870 ± 82.841	89.770 ± 7.630	601.960 ± 28.894	1258.100 ± 56.615	259.830 ± 12.472	192.070 ± 9.219	80.920 ± 3.884	423.890 ± 20.347	92.560 ± 7.868
∑_7_TEQ/∑_16_TEQ(%)	/	94.800 ± 7.204	94.660 ± 8.235	93.760 ± 7.969	94.360 ± 8.209	94.530 ± 8.035	95.500 ± 8.309	94.870 ± 8.064	94.800 ± 8.248	95.590 ± 8.125	93.880 ± 8.168

N.D. stands for none detected; PAHs with * refers to carcinogenic PAHs.

Carcinogenic risk evaluation results of the PAHs in road dust, roof dust, and bare soil inside the ISE and in the surrounding environment were listed in Table 4. The ranges of TILCR for children, adult males, and adult females were (1.061 ± 0.171) × 10^–5^–(7.915 ± 0.579) × 10^–4^, (8.274 ± 0.793) × 10^–6^–(6.175 ± 0.601) × 10^–4^, and (7.486 ± 0.649) × 10^–6^–(5.587 ± 0.512) × 10^–4^, respectively. Road dust exhibited heavier carcinogenic risk than roof dust, and the TILCR of bare soil at S3 was higher than that of road dust and roof dust, indicating potentially heavier pollution in bare soil. Among the different exposure pathways, the order of the carcinogenic risk value was ILCR_derm_ > ILCR_ing_ > ILCR_inh_. ILCR_inh_ did not indicate any carcinogenic risk as the value was lower than 10^–6^. ILCE_ing_ in road and roof dust at S1, roof dust at S2, and bare soil at S2 were between 10^–6^ and 10^–4^, suggesting a low to moderate risk of carcinogenesis, and LCE_derm_ of road and roof dust at S1 and bare soil at S2 exceeded 10^–4^, indicating a higher carcinogenesis risk. For different age groups, ILCR_ing_ and IL_CRinh_ in adults were higher than in children, whereas the ILCR_derm_ value for children was slightly higher than that for adults, indicating that the carcinogenesis risk was increasing with age, but children was easily to suffer from skin contact. Moreover, male ILCR_inh_ and ILCR_derm_ was higher than female, while male ILCR_ing_ was lower than that of female, mainly because of the female lower respiratory rate, lower weight, lower skin contact area, and longer lifetime^25^.

**Table 4:** Carcinogenic risk evaluation results of the PAHs in RD, RF, and BS inside the ISE and in the surrounding environment.

Exposure pathways	Age group	S1		S2		S3		S4		S5	
		Rd	Rf	Rd	Rf	Bs	Rd	Rf	Rd	Rd	Rf
ILCR_ing_	Child	(8.065 ± 0.061) × 10^–6^	(2.308 ± 0.103) × 10^–6^	(1.212 ± 0.227) × 10^–7^	(8.079 ± 0.598) × 10^–7^	(1.685 ± 0.203) × 10^–6^	(3.444 ± 0.149) × 10^–7^	(2.563 ± 0.149) × 10^–7^	(1.081 ± 0.193) × 10^–7^	(5.614 ± 0.429) × 10^–7^	(1.248 ± 0.119) × 10^–7^
	Adult (male)	(1.144 ± 0.125) × 10^–6^	(3.274 ± 0.135) × 10^–6^	(1.719 ± 0.155) × 10^–7^	(1.146 ± 0.149) × 10^–6^	(2.390 ± 0.149) × 10^–6^	(4.886 ± 0.444) × 10^–7^	(3.636 ± 0.149) × 10^–7^	(1.553 ± 0.413) × 10^–7^	(7.964 ± 0.966) × 10^–7^	(1.771 ± 0.416) × 10^–7^
	Adult (female)	(1.170 ± 0.109) × 10^–6^	(3.349 ± 0.444) × 10^–6^	(1.759 ± 0.163) × 10^–7^	(1.172 ± 0.103) × 10^–6^	(2.445 ± 0.249) × 10^–6^	(4.998 ± 0.324) × 10^–7^	(3.719 ± 0.149) × 10^–7^	(1.568 ± 0.416) × 10^–7^	(8.146 ± 0.973) × 10^–7^	(1.811 ± 1.216) × 10^–7^
ILCR_inh_	Child	(3.753 ± 0.023) × 10^–10^	(1.074 ± 0.498) × 10^–10^	(5.641 ± 0.438) × 10^–12^	(3.758 ± 0.211) × 10^–11^	(7.841 ± 0.644) × 10^–11^	(1.603 ± 0.144) × 10^–11^	(1.193 ± 0.197) × 10^–11^	(5.028 ± 0.1) × 10^–12^	(2.613 ± 0.259) × 10^–11^	(5.808 ± 0.318) × 10^–12^
	Adult (male)	(1.570 ± 0.149) × 10^–9^	(4.495 ± 0.416) × 10^–10^	(2.360 ± 0.219) × 10^–11^	(1.573 ± 0.149) × 10^–10^	(3.281 ± 0.119) × 10^–10^	(6.707 ± 0.179) × 10^–11^	(4.991 ± 0.497)) × 10^–11^	(2.104 ± 0.149) × 10^–11^	(1.093 ± 0.106) × 10^–10^	(2.431 ± 0.201) × 10^–12^
	Adult (female)	(1.316 ± 0.135) × 10^–10^	(3.767 ± 0.355) × 10^–10^	(1.978 ± 0.187) × 10^–11^	(1.318 ± 0.216) × 10^–10^	(2.749 ± 0.222) × 10^–10^	(5.621 ± 0.981) × 10^–11^	(4.183 ± 0.416) × 10^–11^	(1.763 ± 0.144) × 10^–11^	(9.161 ± 0.519) × 10^–11^	(2.037 ± 0.106) × 10^–11^
ILCR_derm_	Child	(7.834 ± 0.169) × 10^–4^	(2.242 ± 0.178) × 10^–4^	(1.177 ± 0.198) × 10^–5^	(6.069 ± 0.597) × 10^–5^	(1.637 ± 0.139) × 10^–4^	(3.346 ± 0.961) × 10^–5^	(2.490 ± 0.144) × 10^–5^	(1.050 ± 0.077) × 10^–5^	(5.454 ± 0.444) × 10^–5^	(1.213 ± 0.198) × 10^–5^
	Adult (male)	(6.061 ± 0.249) × 10^–4^	(1.735 ± 0.198) × 10^–4^	(9.109 ± 0.776) × 10^–6^	(5.478 ± 0.446) × 10^–5^	(1.266 ± 0.149) × 10^–4^	(2.588 ± 0.931) × 10^–5^	(1.926 ± 0.179) × 10^–5^	(8.120 ± 0.498) × 10^–6^	(4.219 ± 0.419) × 10^–5^	(9.380 ± 0.799) × 10^–6^
	Adult (female)	(5.470 ± 0.498) × 10^–4^	(1.566 ± 0.149) × 10^–4^	(8.222 ± 0.597) × 10^–6^	(7.926 ± 0.497) × 10^–5^	(1.143 ± 0.138) × 10^–4^	(2.336 ± 0.138) × 10^–5^	(1.739 ± 0.149) × 10^–5^	(7.329 ± 0.619) × 10^–6^	(3.808 ± 0.315) × 10^–5^	(8.466 ± 0.777) × 10^–6^
TILCR	Child	(7.915 ± 0.579) × 10^–4^	(2.265 ± 0.179) × 10^–4^	(1.190 ± 0.295) × 10^–5^	(7.926 ± 0.597) × 10^–5^	(1.654 ± 0.113) × 10^–4^	(3.380 ± 0.213) × 10^–5^	(2.516 ± 0.188) × 10^–5^	(1.061 ± 0.171) × 10^–5^	(5.510 ± 0.444) × 10^–5^	(1.225 ± 0.122) × 10^–5^
	Adult (male)	(6.175 ± 0.601) × 10^–4^	(1.767 ± 0.169) × 10^–4^	(9.281 ± 0.479) × 10^–6^	(6.184 ± 0.588) × 10^–5^	(1.290 ± 0.143) × 10^–4^	(2.637 ± 0.167) × 10^–5^	(1.963 ± 0.179) × 10^–5^	(8.274 ± 0.793) × 10^–6^	(4.299 ± 0.416) × 10^–5^	(9.557 ± 0.792) × 10^–6^
	Adult (female)	(5.587 ± 0.512) × 10^–4^	(1.599 ± 0.167) × 10^–4^	(8.398 ± 0.761) × 10^–6^	(5.595 ± 0.497) × 10^–5^	(1.167 ± 0.112) × 10^–4^	(2.386 ± 0.149) × 10^–5^	(1.776 ± 0.159) × 10^–5^	(7.486 ± 0.649) × 10^–6^	(3.890 ± 0.349) × 10^–5^	(8.647 ± 0.798) × 10^–6^

## Conclusions

To investigate the concentration levels of 16 priority PAHs in road dust and in roof dust inside and in the surrounding region of the ISE and its impact on atmospheric PM, dust and environment PM samples were collected. The results showed that PAH concentrations displayed great variability in dusts. The ∑_16_ PAHs concentrations (in dry weight) were between 0.460 and 46.970 μg/g (avg ± sd 10.892 ± 1.185 μg/g) in road dust, between 0.670 and 17.140 μg/g (avg ± sd 6.751 ± 0.692 μg/g) in roof dust, and 13.990 ± 1.203 μg/g in bare soil. Particle size distribution and PAH distribution of dust samples showed that road dust at low height had a coarser particle size and easily adsorbed high-ring PAHs (PAHs containing four or more rings). For atmospheric PM sites, ∑_16_ PAHs was the highest inside the ISE, followed by sites downwind of the ISE, and the lowest at sites upwind of the ISE. This indicates a greater impact of dust on the atmospheric PM near the ISE. A similar concentration curve was synchronously observed, whereby the most abundant individual PAHs were BbF, Flua, and BkF at atmospheric PM sites and BbF, Chry, and BkF in dusts. BaP/∑_16_ PAHs ranged from 7.03 to 9.62% in atmospheric PM and ranged from 4.44 to 6.28% in dusts, suggesting that PAHs of atmospheric PM should be paid sufficient attention as they may have serious impact on the surrounding atmosphere.

Toxicity analysis revealed that PAHs with four rings, including carcinogenic PAHs, were the dominant pollutants in the studied area, and the Σ_7_TEQ_BaP_ to Σ_16_TEQ_BaP_ ratio ranged from 76.400 to 99.100%. Based on the carcinogenic health risk assessment model recommended by the US EPA, the calculated results showed that skin contact with PAHs was the greatest health risk, followed by ingestion and inhalation. By comparison, road dust presented a greater carcinogenic risk than roof dust, while bare soil may suffer from heavier pollution. Meanwhile, the PAH carcinogenic risk of adults by skin contact and inhalation was higher than that of a child, and male PAH carcinogenic risk was higher than that of female.
